# Post-Release Changes of the Goat’s Rue Rhizobia (*Neorhizobium galegae* bv. *orientalis*) Genomes in the Field: Prospects for Breeding the Symbiotically Effective Strains

**DOI:** 10.3390/ijms27198656

**Published:** 2026-09-28

**Authors:** Nikolay A. Provorov, Nikolay Kurchak, Olga P. Onishchuk, Igor V. Evsyukov, E. P. Chizhevskaya, T. S. Aksenova, A. K. Kimeklis, G. V. Gladkov, A. A. Goncharova, A. G. Pinaev, E. E. Andronov

**Affiliations:** 1 Laboratory of Microbiological Monitoring and Bioremediation of Soils, All-Russian Research Institute for Agricultural Microbiology, Saint-Petersburg 196608, Russia; na.provorov@arriam.ru (N.A.P.); nick.kurchak@yandex.ru (N.K.); o.onishuk@arriam.ru (O.P.O.); igor.v.evsyukov@mail.ru (I.V.E.); chizhewskaya@arriam.ru (E.P.C.); t.aksenova@arriam.ru (T.S.A.); akimeklis@arriam.ru (A.K.K.); grgladkov@arriam.ru (G.V.G.); anutikgon@mail.ru (A.A.G.); apinaev@arriam.ru (A.G.P.); 2 Department of Applied Ecology, St. Petersburg State University, Saint-Petersburg 196608, Russia; 3 Zoological Institute of Russian Academy of Sciences, Saint Petersburg 196608, Russia

**Keywords:** root nodule bacteria (rhizobia), inoculants for legume plants, evolution of symbiosis, adaptation, goat’s rue (*Galega orientalis*), whole genome analysis, insertion elements, tandem duplications, symbiotic effectiveness, nodulation competitiveness

## Abstract

Root nodule bacteria (rhizobia) fixing N_2_ in cooperation with legumes are broadly used for improving production of these plants inoculated with strains selected for high symbiotic activity. These bacteria are characterized by enormous evolutionary plasticity based on genomic rearrangements and horizontal gene transfer. However, this native plasticity is still poorly used for symbiotic improvement of the inoculant strains. In the presented paper, we analyzed genomic structure and symbiotic efficacy in ten clones isolated from nodules formed by the highly effective strain Gr30-13 of goat’s rue rhizobia (*Neorhizobium galegae* bv. *orientalis*) on the host plant (*Galega orientalis*) grown for 1 or 2 years in the soil devoid of aboriginal populations of this rhizobia species. The genome analysis based on nanopore sequencing technology revealed different genomic rearrangements (IS transpositions, tandem duplications) in three clones, which exceed the remaining six clones in symbiotic efficiency. One clone with two tandem duplications in its genome significantly outperformed the initial strain in symbiotic efficiency by more than 35%. We tentatively suggest that the enhanced symbiotic efficiency may be linked to the detected genomic changes; if confirmed, such effects could be described as the “self-improvement” of rhizobia, driven by genomic plasticity and subsequent selection under field conditions. The presented model experiment suggests a new approach for breeding programs using nature-like technology based on the spontaneous genetic variability of symbiotically active rhizobia.

## 1. Introduction

The application of rhizobial inoculants in legume cropping is widespread in sustainable agriculture. A substantial body of evidence shows that rhizobia possess a pronounced evolutionary plasticity that allows them to adapt to both local environmental conditions and the host plant genotypes [1]. A major source of this plasticity is genome rearrangements, to which rhizobia are particularly predisposed owing to their high content of repetitive sequences and mobile genetic elements, especially insertion sequences (IS elements), which can relocate within the genome and thereby disrupt or modify the expression of coding and regulatory sequences [2]. More broadly, the multipartite genome architecture typical of rhizobia, comprising a chromosome together with megaplasmids and chromids, has been shown to be subject to continual structural modification, including the cointegration and excision of entire replicons [3]. Another source of genetic plasticity is represented by mechanisms generating nucleotide-level polymorphism in the corresponding signaling genes, which help to maintain the efficiency of partners’ recognition. For example, concordant patterns of genetic diversity have been reported between the rhizobial *nod*A gene, which participates in the biosynthesis of the Nod-factor signaling molecule, and the plant NFR5 receptor gene responsible for its perception [4]. Taken together, these mechanisms indicate that rhizobia are evolutionarily highly plastic microorganisms with enormous adaptive potential [5,6]. Unfortunately, these mechanisms are still poorly used for generating agronomically valuable inoculant strains.

The hypothesis underlying the presented work is that at least some of the spontaneous genomic changes arising in an inoculant strain after its release into the field are of adaptive value, both for the survival of the strain under the given soil conditions and for the efficiency of its symbiotic interaction with a particular host cultivar. Such changes would be expected to be favored by natural selection acting both at the level of saprophytic (free-living) fitness in the soil and at the level of symbiotic effectiveness and nodulation competitiveness. This expectation is consistent with experimental-evolution studies of rhizobia, in which serial passage of bacteria through legume hosts under controlled conditions, followed by genome resequencing, has been shown to reveal spontaneous mutations that improve nodulation and symbiotic performance over successive generations [7]. In the presented research we tested this hypothesis directly in a field setting, using nodule reisolates of the goat’s rue rhizobia (*Neorhizobium galegae* bv. *orientalis*) inoculant strain Gr30-13 that were recovered from nodules formed during the first and second years after field release, subjected to whole-genome sequencing, and subsequently assessed for their symbiotic properties.

Importantly, research on rhizobial genomic plasticity has only recently become practically feasible on the scale required, owing to the advent of third-generation (long-read) sequencing technologies for microbial genomes. Unlike short-read (shotgun) sequencing, long-read platforms such as those developed by Oxford Nanopore Technologies allow not only the detection of nucleotide-level polymorphism but also the reliable resolution of structural genome variation associated with repetitive sequences and mobile elements, variation that is notoriously difficult to reconstruct correctly from short reads alone [8]. Continuing improvements in nanopore sequencing accuracy and a substantial reduction in cost have made it possible to obtain complete, high-quality bacterial genome assemblies without the need for supplementary short-read data, making the routine sequencing of dozens, or even hundreds, of genomes economically and technically realistic [9].

An essential part of this technology is the availability of a simple and effective method of genomic fingerprinting, capable of distinguishing the progeny of the inoculant strain among the mixture of isolates recovered from field-collected nodules. Numerous genomic fingerprinting techniques for rhizobia have been proposed over the past three decades, including approaches based on repetitive-element PCR and on amplified fragment length polymorphism [10,11]. In the present work, however, we demonstrate the effectiveness, simplicity and reliability of a genomic fingerprinting approach that we have recently developed, based on the multiplex PCR detection of a set of DNA fragments specific to the inoculant strain [12]. Our work suggests the technological opportunity that the present study seeks to exploit: using a model experiment, we outline in general terms a technology by which the spontaneous variation that arises in rhizobia (*N. galegae* bv. *orientalis*) genomes under field conditions can be used for the adaptive improvement of inoculant strains intended for use under the specific field conditions and a specific legume (*G. orientalis*) cultivar. Several reisolates carrying genome structural changes, represented by IS transpositions and tandem repeats, displayed increased symbiotic efficacy relative to the original inoculant strain, suggesting that this approach could be effectively applied to the adaptive improvement of rhizobia inoculant strains. Although confirming the link between the identified genomic rearrangements and changes in symbiotic efficiency requires further detailed study, we believe the observations presented here are sufficient to draw the attention of researchers to what we consider a highly promising avenue in the breeding of the agronomically valuable rhizobial strains.

## 2. Results

### 2.1. Genome Fingerprinting

For genome fingerprint validation, eight Caucasian strains were used as controls in multiplex PCR to determine the specificity of the genomic fingerprint developed for strain Gr30-13. To identify the inoculant strain Gr30-13 in *G. orientalis* nodules from the first and second years of the field experiment, a genomic fingerprint was used. This fingerprint consisted of five pairs of primers for multiplex PCR flanking highly specific fragments of the Gr30-13 genome (3 on the chromosome and 2 on the symbiotic plasmid), which are absent from most *N. galegae* bv. *orientalis* strains. Five fragments ranging in size from 245 to 639 bp were produced with DNA of the Gr30-13 strain in the multiplex PCR reaction (Table 1, Figure 1).

As can be seen from the data presented in Figure 1, strain Gr30-13 and its reisolates exhibit clearly identifiable and strictly identical fingerprints, while 8 control strains isolated in the same region (North Caucasus) as strain Gr30-13 have clear differences from this strain. Further, we designate the strains isolated from the *G. orientalis* nodules of the first and second years and preserving the genomic fingerprint of the strain Gr30-13 as reisolates of this strain. All reisolates showed the same fingerprints. These data suggest the absence in the soil of aboriginal *N. galegae* strains which may interfere with genetic processes occurring in the inoculant strain. Also, no nodules were detected on non-inoculated plants.

### 2.2. Genome Sequencing and Analysis

Whole-genome sequencing was performed on 10 reisolates using Oxford Nanopore technology. The genomes of all reisolates were compared with the original Gr30-13 strain using Pannagram. Pannagram was used in reference-free mode (pangenome alignment of all 11 genomes at once, without anchoring to a single reference), followed by structural-variant calling and a BLAST search of the Gr30-13 genome for the origin of each insertion. Ten rearrangements were found (844–1975 bp) relative to Gr30-13. They are distributed very unevenly: half of the re-isolates carry none. No deletions were revealed. All structural differences are gains located on either chromosome or on chromid harboring *sym* genes. All 10 insertions have homologues in the parental Gr30-13 genome. None came from outside. All insertions were further identified as IS element transpositions, tandem repeats, and nanopore sequencing artifacts (Figure 2, Table 2).

### 2.3. Origin of the Insertions

#### 2.3.1. IS256 Transposition

A homologue in Gr30-13 was found for all insertions, but the events are of two different types (Table 2). IS element transposition (1 event). 8_1_IS and 18_2_IS represent an IS256 family transposase, ISAli6 insertion. In the parental strain Gr30-13 it is already present in nine nearly identical copies (99.93%): three on the chromosome and six on the symbiotic chromid. All nine were independently annotated by Prokka as a transposase of the same family. In Re 8-1 and Re 18-2 the element inserted at a new site, ~0.9 Mb from the nearest original copy. This is the only case of movement over a long distance. An important detail: in pangenome coordinates the insertions in Re 8-1 and Re 18-2 fall into the same interval, suggesting a single event inherited by two re-isolates from a common ancestor, not two independent transpositions. What is more significant here is the fact that these variants were isolated not only from different nodules but also in different years of the experiment. This indicates that in both cases, these were independent infection events, suggesting that the modified strain is more competitive than the original one.

The structure of the element confirms transposition. We inspected the boundaries of the insertion and found the canonical IS element signature, and it is identical in both carriers: (1) target site duplication (TSD) of 8 bp: TTCCTCGA—exactly the length described for the IS256 family (upon insertion the element duplicates 8 bp of the target [13]); (2) terminal inverted repeats (TIR) of 13 bp: GATCGTGTCCCAG—perfect, without a single mismatch; (3) the element proper between the TIRs—1361 bp. In Re 8-1 and Re 18-2 the element inserted at a new site, ~0.9 Mb from the nearest original copy. Because both reisolates share a common ancestor and stem from a single evolutionary event, they should not be treated as independent lines of evidence for an association between this rearrangement and the phenotype.

#### 2.3.2. Tandem Duplications

A total of 9 tandem repeats were identified. For all of them the donor locus lies immediately adjacent to the insertion site (distance 0 in pangenome coordinates): the new copy of the region sits next to the original. Of the 9 tandem repeats, only two, in reisolate Re 20-2, are genuine tandem duplications (Table 2). The remaining identified tandem repeats are not simple copies of adjacent regions, but a combination of direct and inverted copies of the same locus: an exact direct copy at the edges and a diverged inverted copy in the middle. The low identity of the inverted region (68–89% versus 100% for the direct copy) suggests that the inverted repeat at this location has existed for a long time, and the duplication event occurred recently. This is precisely the pattern known to be a technical error in nanopore sequencing, not a biological mechanism [14]. Dot plots for these 7 nanopore artifacts are given in the Supplement (Figures S1–S7).

### 2.4. Structural Effects of Insertions in the Target Sites

After discarding seven insertions that were artifacts of nanopore sequencing, four events remained: two insertions of the IS element in reisolates Re 8-1 and Re 18-2, and two tandem duplications in reisolate Re 20-2. A comparison of the annotations of the original Gr30-13 genome and the genomes of the corresponding reisolates at the insertion sites is shown in Figure 3.

As can be seen from the data presented in Figure 2, a tandem duplication of 1040 bp 20_2_TD_1 in isolate Re 20-2 occurred on the chromosome inside the *ngc*G gene, and led to the appearance in the annotation of a new chimeric reading frame consisting of a 5’ fragment of the *ngc*G gene and a 3’ fragment of the *ugp*C gene. Both fragments of the chimeric gene retained their original reading frames in this annotation. According to the UniProt database, the identified *ngc*G gene and its chimeric versions correspond to the gene from the strain *Neorhizobium galegae* bv. *orientalis* str. HAMBI 540, encoding a permease (A0A068SQN8) that transports sugars into the cell and is located within the operon of ABC carbohydrate transporter proteins. It is worth noting that the start site of the *ngc*G gene varies greatly among different members of the tribe Rhizobiaceae.

The second tandem duplication 20_2_TD_2 (844 bp) occurred on the symbiotic chromid and affected genes *bgl*B and *arg*E. According to the genomic annotation of reisolate Re 20-2, this tandem duplication resulted in the appearance in the annotation of two new reading frames *arg*E2 and *bgl*E2, the first of which is a 3′ gene fragment, and the second a 5′ gene fragment. As with the tandem duplication on the chromosome of reisolate Re 20-2, both retained the reading frame of the original genes in the annotation. *arg*E encodes acetylornithine deacetylase (A0A068SZ33). We can indirectly link the possible consequence of this insertion to the distant homology between the *arg*E gene and the chitooligosaccharide deacetylase gene *nod*B, which also contains a polysaccharide deacetylase domain belonging to Carbohydrate Esterase Family 4 (CE4) [15]. For the future research we plan a specific testing via mutation and transcriptomic analyses of the roles implemented by *bgl*B and *arg*E genes in control of *N. galegae* symbiotic efficiency.

The third event is an identical insertion of an IS element into the chromosome of reisolates Re 8-1 and Re 18-2. The insertion occurred within the gene *gcv*A and, as indicated by the annotations of the reisolates, divided it into two reading frames. Interestingly, as in the two previous cases, both retained their original reading frame in this annotation. However, the two newly introduced genes are annotated differently: *dcd*C and *hdf*R. All three genes are annotated as glycine cleavage system transcriptional activators (A0A068SSI4), with *hdf*R inheriting the HTH lysR-type domain responsible for DNA binding from *gcv*A. *dsd*C loses this domain but inherits the regulatory C-terminal domain, which forms a co-inducer-binding cleft [16]. The literature shows that disruption of the glycine cleavage system leads to nodule formation in *Sinorhizobium fredii* USDA257 on one of the soybean cultivars [17] due to an unknown mechanism.

### 2.5. Symbiotic Properties of Reisolates

An important aspect of this study is that nine reisolates were tested for symbiotic efficacy (the ability to increase goat’s rue plant mass through symbiotic nitrogen fixation) in a sterile tube test (the isolate Re 19-2 failed the test). The experiment was performed in six biological replicates (individual host plants) for each reisolate, along with controls (the original strain Gr30-13 and a control without inoculation). The results are shown in Figure 4.

All studied clones exceed the initial strain in symbiotic efficiency (ability to enhance the plant shoot mass) on average by 20.76 ± 3.68%; in one strain (Re 20-2) the increase of symbiotic efficacy was significant at *p* < 0.05 (35.23%).

## 3. Discussion

The aim of the present study was to demonstrate, within the framework of a small-scale model experiment, the prospects for detecting and practically exploiting adaptive genomic changes in goat’s rue rhizobia (*N. galegae* bv. *orientalis*) inoculant strains. The central hypothesis underlying this experiment suggests that cooperative impacts of genomic rearrangements and of host-induced natural selection operate in the absence of local *N. galegae* strains that may recombine and compete with the inoculant for host nodulation.

A considerable body of literature describes examples in which rhizobial strains acquire new adaptive traits through a range of evolutionary mechanisms, including genome rearrangements, horizontal gene transfer and nucleotide substitutions [2,3,18]. As this evidence indicates, changes of this kind can potentially be of considerable agronomic value and may suggest the adaptive strategies by which inoculant strains, selected in different regions and using diverse hosts, become adapted to novel plant genotypes grown under local edaphic conditions.

The presented research revealed the changes in genomic structure and symbiotic efficacy in nine clones isolated from nodules formed by the highly effective *N. galegae* strain Gr30-13 on the host plant (*Galega orientalis*) grown for 1 or 2 years in the soil devoid of aboriginal populations of this rhizobial species. All studied clones exceed the initial strain in symbiotic efficiency (ability to enhance the plant shoot mass) on average by 20.76 ± 3.68%. According to the rigorous statistical test (see Materials and Methods), in one strain (Re 20-2) the increase of symbiotic efficacy was significant at *p* < 0.05 (35.23%). Other re-isolates (Re 8-1, Re 18-2), together with Re 20-2, remain of interest for the “second round” of field experiments on spontaneous variability, given their detected genomic rearrangements.

The genomic sequencing based on ONT technology revealed different genomic rearrangements (IS transpositions, tandem duplications) in three clones. These clones exceed the remaining six non-rearranged clones in symbiotic efficiency: average increases over the initial strain for these groups of clones are 31.14 ± 2.12% and 15.57 ± 3.93%, respectively (*p* < 0.05) (Table 3). No difference was revealed in symbiotic efficiency between groups of clones isolated in the 1st and 2nd years of plant growth: for these groups, average increases over the initial strain are 22.36 ± 3.35% and 18.89 ± 4.55%, respectively (*p* > 0.10). These data can be cautiously interpreted in terms of “symbiotic self-improvement” in rhizobia, driven by genomic plasticity and subsequent selection under field conditions. Importantly, the improvement in symbiotic efficiency resulting from IS insertions—which typically cause target gene inactivation—aligns with previously published results in alfalfa rhizobia (*Sinorhizobium meliloti*). In those studies, the capacity to increase plant biomass was enhanced by Tn5 insertions into genes identified as negative regulators of symbiosis [19].

If a functional link does indeed exist between the observed genome rearrangements and symbiotic efficacy—a possibility requiring further validation—our findings suggest an intriguing implication: modifications of this type are exceedingly difficult to engineer deliberately, since the efforts of genetic engineers are generally directed at adding new genes or strengthening the regulation of the existing ones. Deliberately engineering the formation of a chimeric gene through tandem duplication, specifically in order to increase symbiotic efficiency, is probably scarcely conceivable by rational design. This suggestion is consistent with a substantial body of work on bacterial gene duplication and amplification, which has shown that such events generate extensive genetic variation and provide the raw material from which genuinely new gene functions can arise through subsequent divergence [20,21,22]. It is also consistent with experimental-evolution studies of rhizobia, in which genome resequencing of bacteria passaged repeatedly through legume hosts has revealed spontaneous mutations that improved symbiotic performance without having been designed by the experimenters [23]. A great potential of the plant-induced selection for modification of the rhizobia symbiotic properties is evident from the reorganization of the extracellular pathogen *Ralstonia solanacearum* into an intracellular beneficial legume symbiont via serial cycles of legume-bacterium co-cultures [24].

Thus, in the present study we are speculating about the possibility of suggesting a new approach for using the spontaneous variability of rhizobia to adapt inoculant strains to local conditions. If supported by an extended analysis of rhizobia variability, it may be developed into a technology which involves the following elements: (1) inoculation of the legume host plant by the effective strain in the absence of local rhizobia that may compete or recombine with the inoculant; (2) isolation of the inoculant strain derivatives from the nodules and their testing for genomic rearrangements; (3) search for highly effective re-isolates among the rearranged strains.

The potential of the proposed approach is supported in the present work by our recently developed robust genomic fingerprinting approach [12], and by advances in long-read sequencing technology, which have made whole-genome sequencing of dozens of re-isolates practically feasible [9]. The proposed technology represents an agricultural application of natural evolutionary processes, directed at improving the agronomic potential of the symbiotic system. Clearly, such technologies will not involve genetic engineering procedures, and therefore satisfy the requirements for genetic safety of “green” agro-technologies [24]. In addition, the data obtained in the course of these experiments will help to understand the fundamental genetic mechanisms of control of symbiotic efficiency that require extended genetic, molecular and bioinformatic research [5].

## 4. Materials and Methods

### 4.1. Strain Isolation and Vegetation Tests

The strains of *N. galegae* bv. *orientalis* Gr16-10, Gr17-3, Gr38-4, Gr8-7, Gr33-6, Gr26-3, Gr26-4, Gr33-3, Gr30-13 were isolated earlier from the nodules of wild-growing *Galega orientalis* in the North Caucasus in 2019 [25]. Re-isolates from nodules of *Galega orientalis* cv. Yalginsky grown in an experimental soil plot of ARRIAM were obtained from plants of 1st (Re 6-1, Re 7-1, Re 8-1, Re 9-1, Re 10-1) and 2nd (Re 16-2, Re 17-2, Re 18-2, Re 19-2, Re 20-2) year of vegetation.

The field experiment with *G. orientalis* cv. Yalginsky and *N. galegae* bv. *orientalis* strain Gr30-13 was conducted on a fallow sod-podzolic soil area on the ARRIAM institute’s territory (N 59°44′20.42″, E 30°25′45.03″). Sterilized *G. orientalis* seeds treated with an aqueous suspension of the strain Gr30-13 were sown in the plot. Rhizobia were isolated from plant nodules formed in the first and second years of the experiment. The sampled *Galega orientalis* plants and roots with nodules were brought to the laboratory and nodules were cut from the roots and washed for 2 min in 70% ethanol, 4 min in 4% Ca(OCl)_2_, and three times in sterile water. The nodules were crushed, suspended in 100 µL of liquid TY medium [26], and streaked out on TY agar. Single colonies were picked and restreaked. Rhizobial isolates that retained the genomic fingerprint (see below) of the original strain Gr30-13 are hereafter referred to as “reisolates” of the strain Gr30-13.

The symbiotic properties of *N. galegae* bv. *orientalis* strains were studied on *G. orientalis* cv. Yalginsky grown for 30 days under aseptic conditions in 60 mL glass tubes with vermiculite and mineral N-free Krassilinikov-Korenyako medium which contains per 1 L of distilled water: K_2_HPO_4_ 1 g, MgSO_4_ × 7H_2_O 1 g, Ca_3_(PO_4_)_2_ 0.2 g, FeSO_4_ traces (pH = 7.0–7.2) [27]. Each tube contains 5 g of vermiculite (particles 2–4 mm) and 10 mL of Krassilinikov-Korenyako medium.

Ten bacterial strains were analyzed: the parental strain GR30-13 and nine re-isolates (Re 6-1, Re 7-1, Re 8-1, Re 9-1, Re 10-1, Re 16-2, Re 17-2, Re 18-2, Re 20-2). Each strain was tested in 6 biological replicates (individual plants); 1 mL of 10^8^ cells/mL of bacterial suspension was added per tube. The response variable was the shoot dry mass (mg) of *G. orientalis* seedlings (strain Re 19-2 failed this test due to a technical error).

### 4.2. Statistical Analysis

Eleven groups were analyzed: the reference group Gr30-13, nine Re lines (Re 6-1, Re 7-1, Re 8-1, Re 9-1, Re 10-1, Re 16-2, Re 17-2, Re 18-2, Re 20-2) and a control group. Each group comprised 6 biological replicates (the control group had 4 valid values; two missing entries, marked as nd, were excluded from the analysis).

Assumptions for parametric testing were checked using the Shapiro–Wilk test (*scipy.stats.shapiro*; normality of residuals; W = 0.966, *p* = 0.077) and Levene’s test with median centering (*scipy.stats.levene*, center = “median”; homogeneity of variances; *p* = 0.566). Overall between-group differences were assessed by one-way ANOVA (*scipy.stats.f_oneway*; F = 8.05, *p* < 0.001). Pairwise comparisons of each group against the reference group Gr30-13 were performed with Dunnett’s test (*scipy.stats.dunnett*, two-sided), which controls the family-wise error rate (FWER) for multiple comparisons against a single reference group. Differences were considered significant at *p* < 0.05; significance levels on the chart are denoted by asterisks: * *p* < 0.05, *** *p* < 0.001; non-significant comparisons are left unmarked. Analyses were performed in Python 3.11.15 using pandas 2.3.3 [28], NumPy 2.4.6 [29], SciPy 1.17.1 [30] and Matplotlib 3.11.1 [31] for visualization.

Group means (three strains with genomic changes versus 6 strains without changes and reisolates of the first year versus the second year) were compared with Welch’s unequal-variance *t*-test (*scipy.stats.ttest_ind*, equal_var = False). Assumptions for parametric testing were checked using the Shapiro–Wilk test. The standardized effect size was expressed as Cohen’s d based on the pooled standard deviation, and the difference between group means is reported with its 95% confidence interval. Tests were two-sided and differences were considered significant at *p* < 0.05. All analyses were performed in Python 3.11.15 with NumPy 2.4.6, pandas 2.3.3 and SciPy 1.17.1.

### 4.3. DNA Isolation

For Nanopore sequencing, rhizobial DNA was isolated using a standard method [32]. DNA purity and integrity were checked by electrophoresis and on an Implen NanoPhotometer (Implen, München, Germany). DNA for the rapid mPCR test was prepared by fast lysis of a crushed nodule or a colony from agar in 20 µL (0.25% SDS, 0.25 mM NaOH) with 1 min heating (95 °C) followed by dilution with water to 200 µL and centrifugation (5 min, 14,000 rpm).

### 4.4. Multiplex PCR for Gr30-13 Strain Identification

The primer design was performed as described previously [12]. Briefly, the whole genome of the strain Gr30-13 was aligned with the whole genome of the reference strain *N. galegae* bv. *orientalis* HAMBI540 (GCA_000731315.1). Ten genome fragments longer than 1000 bp, unique to the strain Gr30-13, were selected and concatenated. The concatenate was nBLASTed against all available *N. galegae* bv. *orientalis* genome contigs in GenBank. Five 1000–2000 bp fragments of the concatenate (three from the chromosome and two from the symbiotic plasmid) with the lowest representation of homologues in GenBank were selected for multiplex PCR primer design. Finally, five primer pairs flanking unique DNA fragments of the Gr30-13 strain genome ranging in length from 245 to 639 bp were designed (Table 1).

A standard PCR protocol was used: a reaction contained 1 U of Encyclo polymerase (Evrogen, Moscow, Russia), a double portion of dNTPs, and 5 pmol each of 10 primers in a final volume of 15 µL. DNA samples were used either as pure DNA (for comparing native strains with Gr30-13) or as rapid lysates (for reisolate screening). 1–5 ng of pure DNA was added to the reaction, as was 1 µL of lysate for the rapid lysates. The thermal profile used was: 35 cycles (95 °C—30 s, 55 °C—30 s, 72 °C—1 min). Amplified DNA fragments were separated in a 2% agarose gel with 0.5x TAE buffer, stained with ethidium bromide and documented with a digital camera under UV illumination.

### 4.5. Genome Sequencing, Assembly and Annotation

The quality and quantity of DNA for whole-genome sequencing were assessed using gel electrophoresis, nanophotometric measurements on an Implen instrument, and fluorometric measurements on a Qubit 4.0 instrument using the dsDNA High Sensitivity Assay Kit and dsDNA Broad Range Assay Kit (Invitrogen, Carlsbad, CA, USA).

Genome sequencing was performed on a MinION sequencer (Oxford Nanopore Technologies, Oxford, UK). For Nanopore sequencing, library preparation was carried out according to the manufacturer-recommended Native barcoding genomic DNA protocol, using the SQK-LSK109 Ligation Sequencing Kit and the EXP-NBD104 and EXP-NBD114 Native Barcoding Kits (Oxford Nanopore Technologies, Oxford, UK). The libraries were sequenced on an R9.4.1 flow cell according to the manufacturer’s instructions. Genome assemblies from Oxford Nanopore Technologies (ONT) reads were generated using a standard long-read pipeline: raw signal data were basecalled with Dorado v1.0.2 [33], assembled de novo with Flye v2.8.1 [34], and the resulting contigs were polished with Medaka 1.11.3 [35] to improve consensus accuracy. Circular contigs were oriented and standardized with dnaapler v1.4.0 [36], and genome annotation was performed using Prokka v1.14.6 [37].

The genomes were deposited in GenBank under BioProject accession number PRJNA1510807.

### 4.6. Genome Analysis

Whole-genome comparison of the original strain Gr30-13 and its ten re-isolates was performed with Pannagram [8] in reference-free mode: all 11 assemblies were aligned simultaneously, with no genome designated as the reference, which yields a single system of pangenome coordinates and avoids reference-specific bias. The chromosome and the symbiotic chromid were treated as separate replicons. Structural variants were called from the resulting alignment, with insertion/deletion polarity defined relative to Gr30-13 as the ancestral strain. Variants longer than 100~bp were retained for further analysis; shorter indels (1–10 bp) are indistinguishable from sequencing errors in nanopore assemblies and were not considered. The origin of each insertion was established by a BLAST search against the Gr30-13 genome.

## 5. Conclusions

The model experiment presented here suggests potential avenues for developing nature-inspired technologies that leverage the genetic variability of symbiotically active rhizobia in breeding programs, according to the following scheme: (1) inoculation of the legume host plant by the effective strain in the absence of local rhizobia that may compete or recombine with the inoculant; (2) isolation of the inoculant strain progeny from the nodules and their testing for genomic rearrangements; (3) search for highly effective re-isolates among the rearranged strains. This technology is based on the hypothetical phenomenon of “symbiotic self-improvement” in rhizobia, which is driven by genomic plasticity and subsequent selection under field conditions.

## Figures and Tables

**Figure 1:**
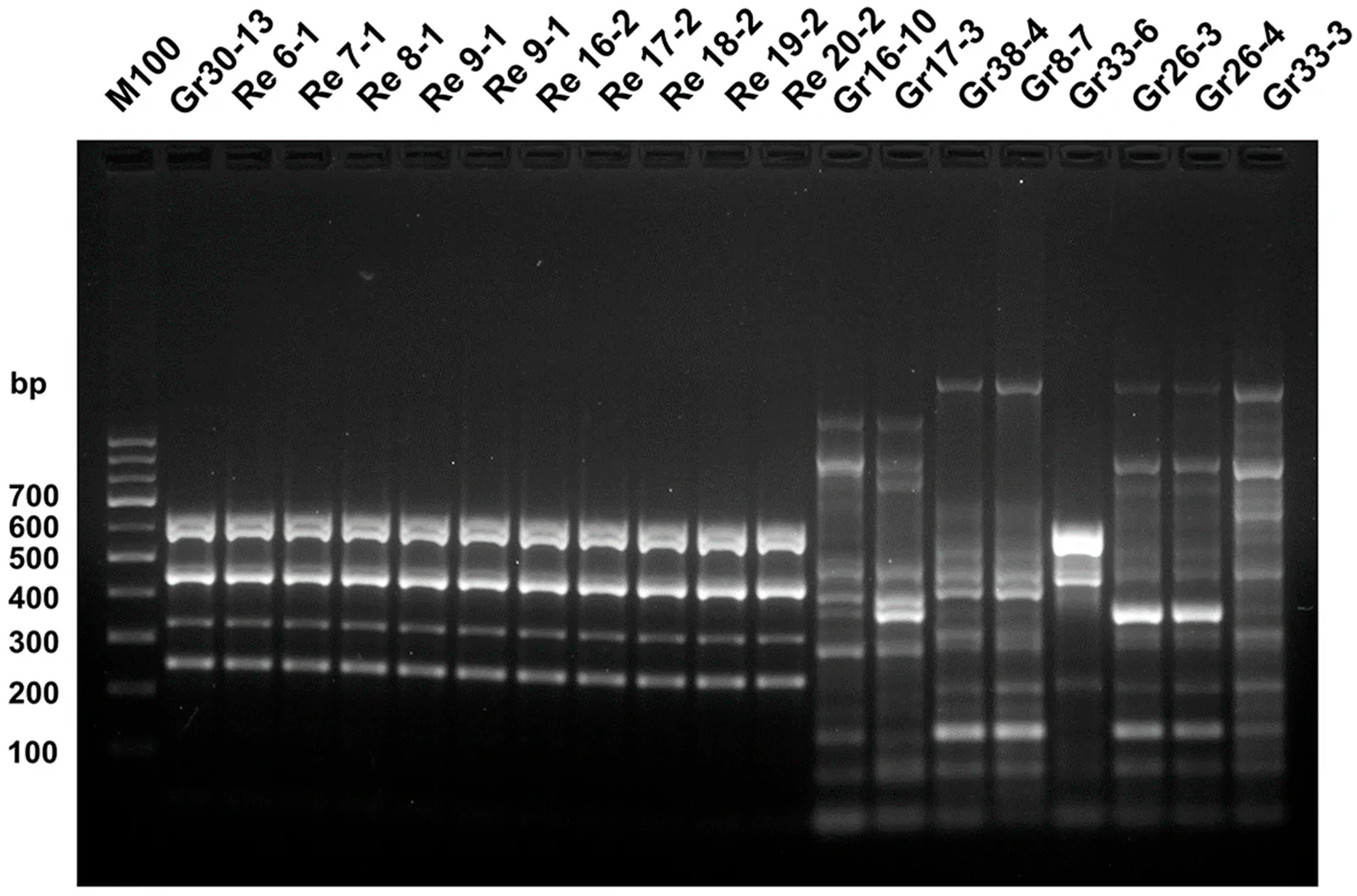
Genomic fingerprinting of strain Gr30-13, its 10 reisolates and 8 native strains of *N. galegae* bv. *orientalis* isolated in the same Caucasian region as Gr30-13.

**Figure 2:**
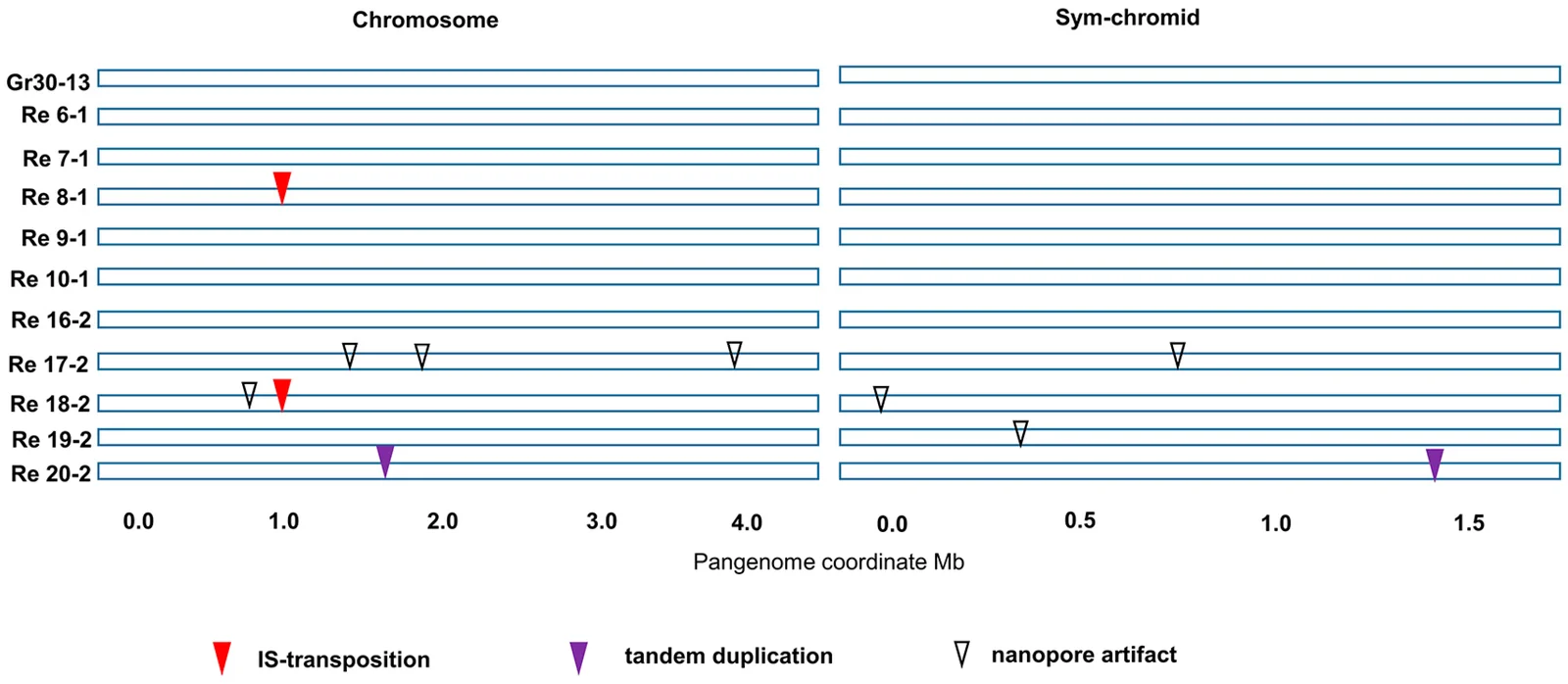
Schematic representation of genomic rearrangements detected in reisolates of Gr30-13.

**Figure 3:**
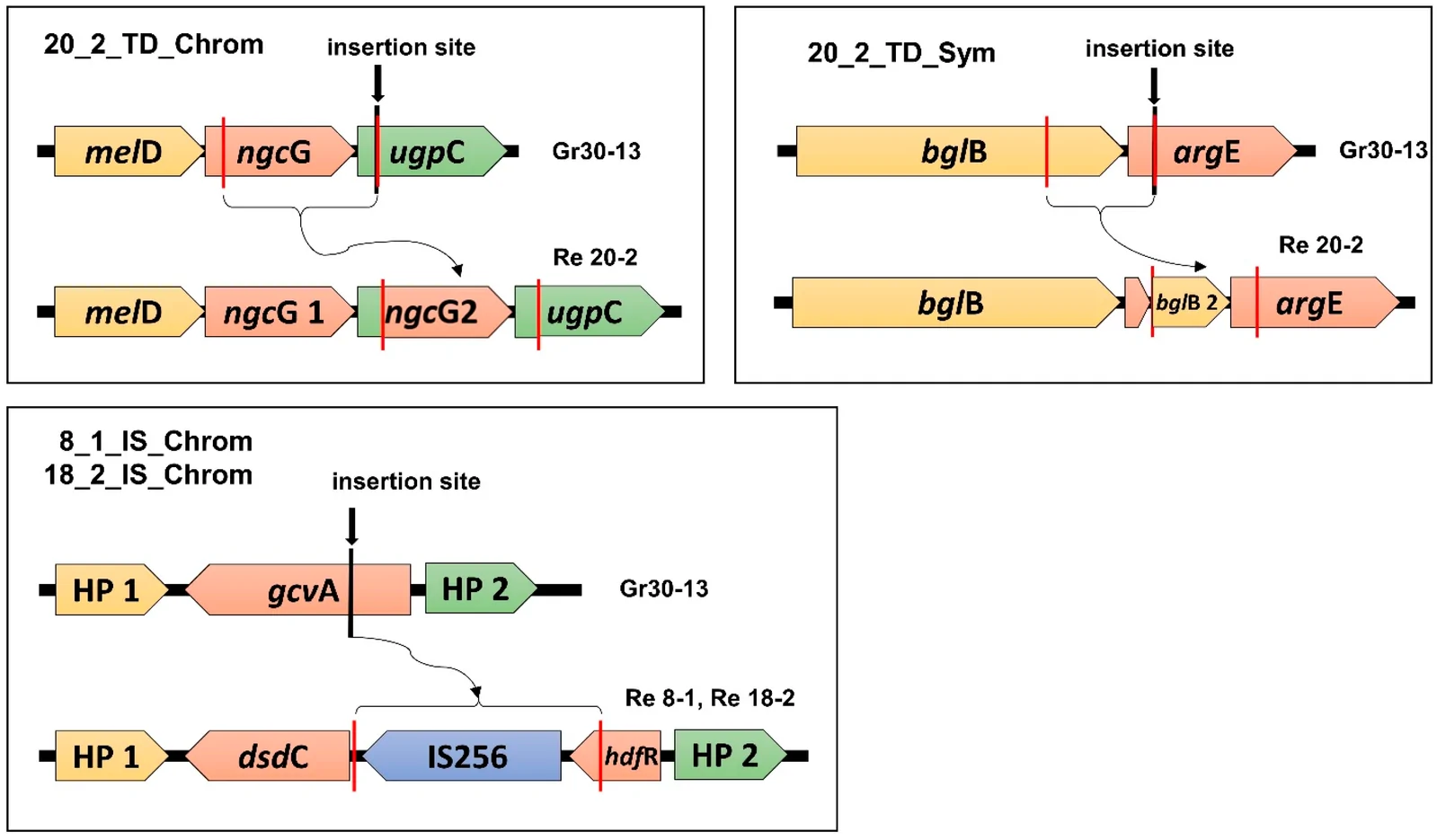
Structural effects of tandem duplications and IS-transpositions in Gr30-13 reisolates: tandem duplications (20_2_TD_Chrom and 20_2_TD_Sym) and IS-transposition (8_1_IS_Chrom and 18_2_IS_Chrom).

**Figure 4:**
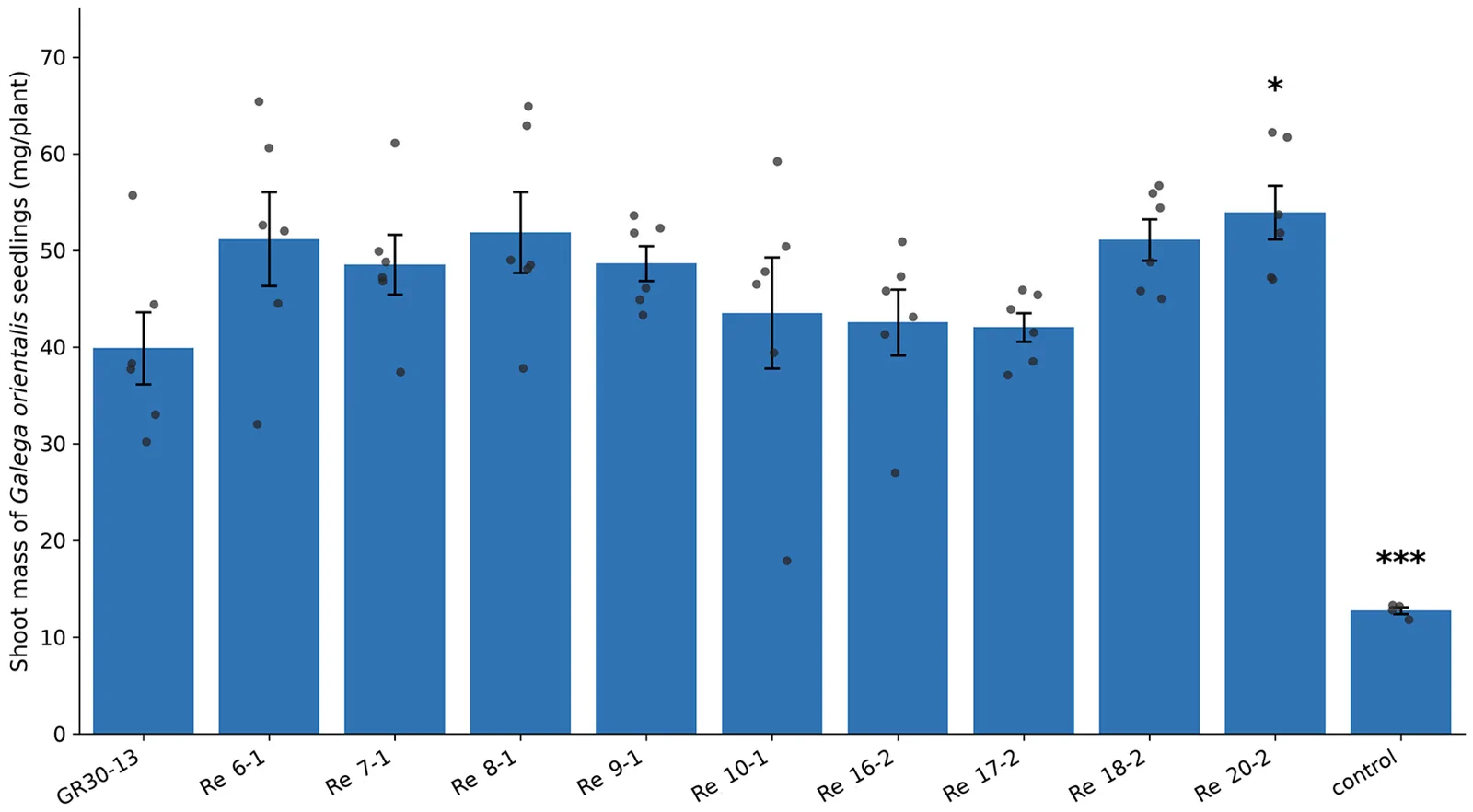
Symbiotic efficiency of the Gr30-13 strain and its reisolates. Bars indicate standard errors. Asterisks indicate a significant difference from the treatment with the original strain Gr30-13 (* *p* < 0.05; *** *p* < 0.001).

**Table 1:** Primers for multiplex fingerprinting of the strain *N. galegae* Gr30-13 (Ch—chromosome, sym—chromid harboring *sym* genes).

N	Fragment Size, bp	Forward Primer	Reverse Primer	Genome Location	Replicon
1	245	GAAAGCAGCGACCGGAGGTT	GAAGAGATGGCCGGGCACAT	130538-130782	Ch
2	322	GAGCAGCCGTCGTAGATCAT	CGATGGAGAACGCAAATTGA	1047729-1048050	Ch
3	450	AATGGGAACAAAAGAAGAAC	GACTCGAAGATAAACTCTAACG	1591680-1592129	Ch
4	600	TTGAGGAAGTCGCTGACAAG	GCCAGATGGCTCGTTTATCG	4794576-4795175	sym
5	639	GTCGCGGATCTCCATGATGT	CGGCATCAATCGAGAACAAG	5765100-5765738	sym

**Table 2:** Insertions detected in genomes of reisolates of the strain *N. galegae* Gr30-13.

ID	Length bp	Strain: Insertion Position	Site in GR30-13 (After)	Donor Locus in GR30-13	Mechanism	Donor Annotation
8_1_IS 18_2_IS	1369	**Re 8-1:** 1,383,143–1,384,511 **Re 18-2:** 956,466–957,834;	955,311	CHROM: 44,639–45,999 (+8 more copies)	IS transposition	IS256 family transposase ISAli6
17_2_TD_1	1333	**Re 17-2:** 1,404,668–1,406,000	1,404,647	CHROM: 1,403,894–1,404,647	tandem duplication NP_Art ^1^	hypothetical protein; 4,4′-diapolycopene aldehyde oxidase
17_2_TD_2	1263	**Re 17-2:** 1,884,738–1,886,000	1,883,368	CHROM: 1,882,986–1,883,699	tandem duplication NP_Art	hypothetical protein; tyrosine—tRNA ligase
17_2_TD_3	873	**Re 17-2:** 3,926,001–3,926,873	3,923,346	CHROM: 3,923,106–3,923,554	tandem duplication NP_Art	L-hydantoinase; hydantoin racemase
18_2_TD_1	1138	**Re 18-2:** 745,863–747,000	745,846	CHROM: 745,272–745,911	tandem duplication NP_Art	two hypothetical proteins
20_2_TD_1	1040	**Re 20-2:** 1,616,961–1,618,000	1,616,947	CHROM: 1,616,948–1,617,986	tandem duplication	NgcG (diacetylchitobiose transport); UgpC
18_2_TD_2	1975	**Re 18-2:** 293,116–295,090	9257	SYM: 8953–9445	tandem duplication NP_Art	GsiC, glutathione transport permease
17_2_TD_4	1241	**Re 17_2:** 1,150,001–1,151,241	760,165	SYM: 759,809–760,505	tandem duplication NP_Art	putative CoA-transferase
19_2_TD_1	996	**Re 19-2:** 858,098–859,093	363,723	SYM: 363,724–364,518	tandem duplication NP_Art	hypothetical protein
20_2_TD_2	844	**Re 20-2:** 1,179,001–1,179,844	1,406,931	SYM: 1,406,837–1,407,670	tandem duplication	acetylornithine deacetylase; β-glucosidase

^1^ Nanopore artifact.

**Table 3:** Genomic rearrangements and symbiotic efficiency in *Neorhizobium galegae* strains.

Strains	Genomic Rearrangements	Shoot Dry Mass of *Galega orientalis*		
		mg/Plant	Increase (%) over Non-Inoculated Control	Increase (%) over Initial Strain
Gr30-13	No	39.88	312.1	0
Nodule reisolates	No (6 clones) *	46.09 ± 1.57	360.6 ± 12.3	15.57 ± 3.93
	Present (3 clones) *	52.30 ± 0.85	409.2 ± 6.6	31.14 ± 2.12

* For both groups of clones, means with standard errors are presented. For all tested parameters, groups of clones with and without genomic rearrangements differ at *p* < 0.05.

## Data Availability

The data is available in GenBank under BioProject accession number PRJNA1510807.

## Supplementary Materials

The following supporting information can be downloaded at https://www.mdpi.com/article/10.3390/ijms27198656/s1.
