# Supplementary material for: Post-Release Changes of the Goat’s Rue Rhizobia (Neorhizobium galegae bv. orientalis) Genomes in the Field: Prospects for Breeding the Symbiotically Effective Strains
Source: Int J Mol Sci. 2026 Sep 28;27(19):8656. doi: 10.3390/ijms27198656 (PMC13664976; doi:10.3390/ijms27198656)
Supplement: Supplementary file 1 [file ijms-27-08656-s001.zip › ijms-4531083-supplementary.pdf]

Supplementary meterials

Post-Release Changes of the Goat's Rue Rhizobia (*Neorhizobium galegae* bv. *orientalis*) Genomes in the Field: Prospects for Breeding the Symbiotically Effective Strains

Nikolay A. Provorov, Nikolay Kurchak, Olga P. Onishchuk, Igor V. Evsyukov, E. P. Chizhevskaya, T.S. Aksenova, A.K. Kimeklis, G.V. Gladkov, A.A. Goncharova, A.G. Pinaev and E.E. Andronov *

Dot plots demonstrating nanopore artifacts in whole genome reisolates of the strain Gr30-13.

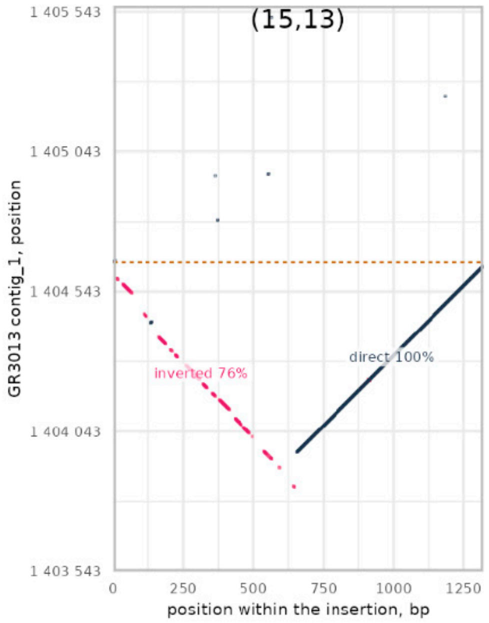

Fig.S1_1. 17_2_TD_1 — a direct copy of 677 bp at 100% and an inverted one of 722 bp at 76%. The two halves of the insertion come from the same region but in opposite orientations.

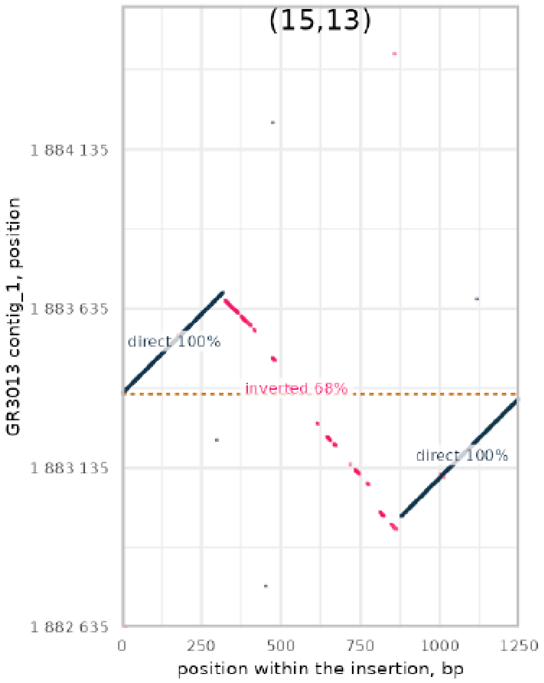

Fig_S1_2. 17_2_TD_2 — two direct blocks (up to 383 bp, 100%) and a long inverted one of 632 bp at 68%. The lowest identity of an inverted part in the whole set.

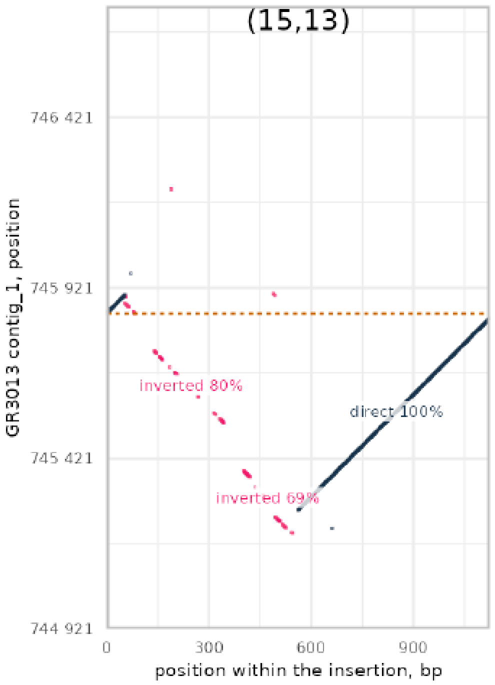

Fig_S1_3. 18_2_TD_1 — an exact direct copy of 575 bp at 100% along the edge and two inverted blocks (up to 226 bp, 80%) in the middle. One of the three insertions whose

donor covers only about half the length.

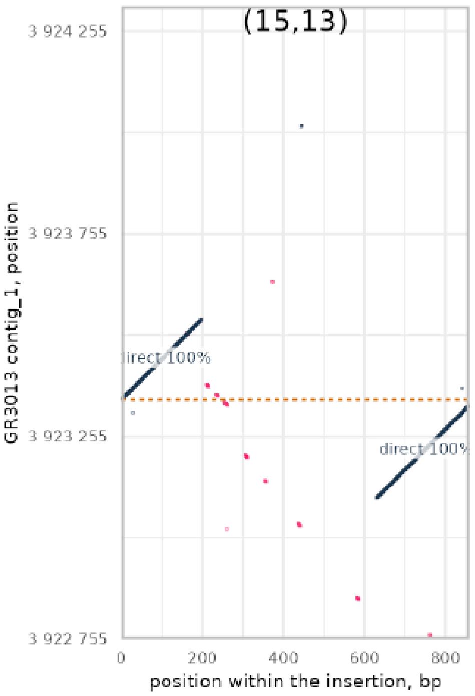

Fig_S1_4. 17_2_TD_3 — two direct blocks (up to 241 bp, 100%); the inverted components here are short, 61–75 bp at 75–89%. The donor covers only 51% of the length — the lowest coverage in the set.

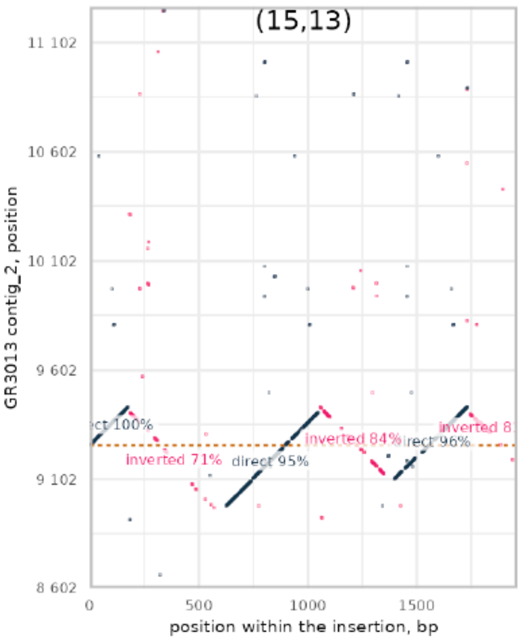

Fig_S1_5. 18_2_TD_2 — the longest and most complex insertion: three direct blocks (up to 468 bp, 100%) and three inverted ones (up to 459 bp, 84%). The terminal 77 bp are the only fragment in the whole set for which no homologue was found in GR30-13.

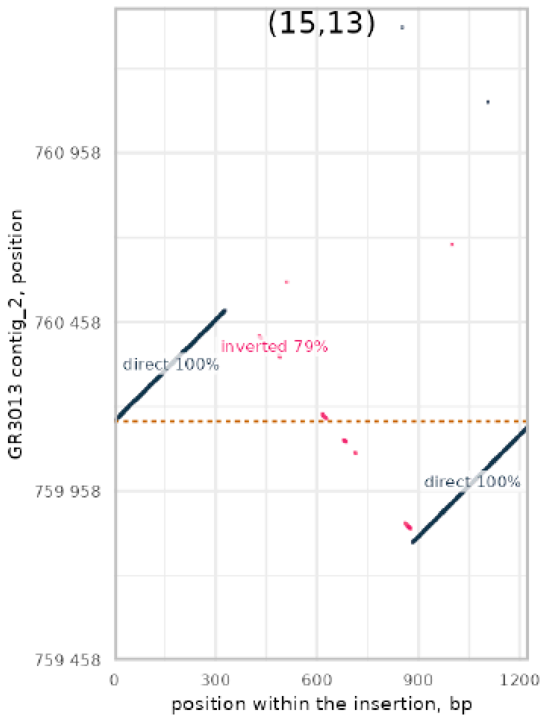

Fig_S1_6. 17_2_TD_4 — two direct blocks (up to 357 bp, 100%) and an inverted one of 145 bp at 79%, plus shorter inverted fragments.

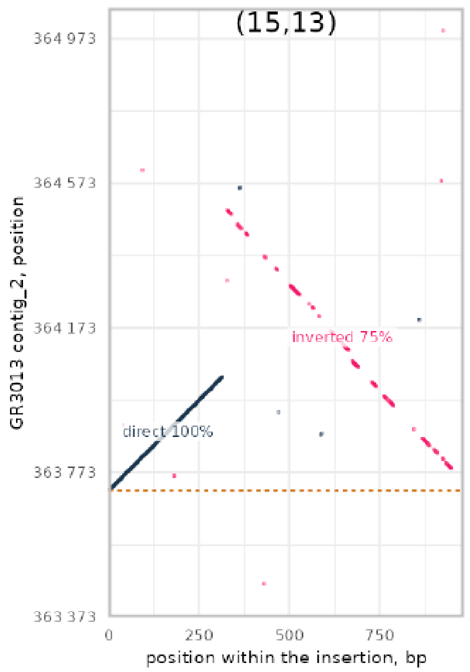

Fig_S1_7. 19_2_TD_1 — a direct block of 328 bp at 100% and a long inverted one of 753 bp at 75%: here the inverted part is larger than the direct one.
